# Effects of Mono‐ and Dicapsaicinoid Functionalization on the Physicochemical Properties and Antimicrobial Photodynamic Activity of Protoporphyrin IX

**DOI:** 10.1002/cmdc.70475

**Published:** 2026-09-07

**Authors:** Ieda Vieira da Cunha, Gabriel Guimarães Calefi, Nagela Bernadelli Sousa Silva, Júlia Gomes Teixeira, Marcos Eduardo Gomes do Carmo, Paula Derksen Macruz, Eduardo Jorge Pilau, Antônio Otávio de Toledo Patrocínio, Carlos Henrique Gomes Martins, Celso de Oliveira Rezende Júnior, Tayana Mazin Tsubone

**Affiliations:** ^1^ Institute of Chemistry Federal University of Uberlândia Uberlândia Minas Gerais Brazil; ^2^ Institute of Biomedical Sciences Federal University of Uberlândia Uberlândia Minas Gerais Brazil; ^3^ Department of Chemistry State University of Maringá Maringá Paraná Brazil; ^4^ Department of Chemistry Federal University of Juiz de Fora Juiz de Fora Minas Gerais Brazil

**Keywords:** antimicrobial photodynamic therapy, multidrug resistance, porphyrin

## Abstract

Antimicrobial photodynamic therapy (aPDT) is a promising alternative for combating multidrug‐resistant bacteria and overcoming limitations of conventional antimicrobial treatments. To improve aPDT performance, two Protoporphyrin IX (PpIX) derivatives functionalized with one or two capsaicin fragments were synthesized and biologically evaluated. Their physicochemical, photophysical, and antimicrobial properties were assessed against susceptible and resistant Gram‐positive (*Staphylococcus aureus*) and Gram‐negative (*Escherichia coli*) strains. Mono‐ and disubstituted capsaicinoid‐functionalized derivatives were isolated in 17% and 40% yields, respectively. Structural modification induced minor photophysical changes compared to PpIX, including slightly reduced singlet oxygen generation and photostability. Aggregation studies revealed that PpIX‐capsaicin **1** exhibited a lower tendency to aggregate, whereas PpIX‐capsaicin **2** showed persistent aggregation with coexistence of H‐ and J‐type species. A significant increase in lipophilicity (Log *P*) was observed only for PpIX‐capsaicin **2**. Photoinactivation assays demonstrated superior efficacy of PpIX‐capsaicin **1** against *S. aureus*, achieving ~3‐log reduction, while limited activity was observed against *E. coli*, likely due to its complex cell envelope. In contrast, PpIX‐capsaicin **2** showed no significant photoactivity. Overall, these findings demonstrate that increased functionalization does not necessarily improve photodynamic performance, highlighting the importance of balanced lipophilicity and aggregation control in photosensitizer design.

## Graphical abstract

Capsaicin functionalization of Protoporphyrin IX influences aggregation behavior, lipophilicity, and photodynamic activity. A monosubstituted derivative exhibits enhanced antimicrobial photoinactivation of *Staphylococcus aureus*, whereas increased functionalization does not improve efficacy. The findings highlight the importance of balancing lipophilicity and aggregation to optimize photosensitizer performance for antimicrobial photodynamic therapy.

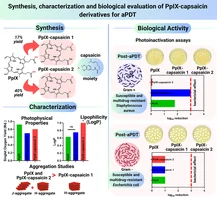

## 1 Introduction

Antimicrobial resistance represents a major global health challenge, compromising the effectiveness of conventional antibiotics and increasing the prevalence of multidrug‐resistant (MDR) bacterial infections [1, 2]. In this context, the development of alternative therapeutic strategies is crucial, and antimicrobial photodynamic therapy (aPDT) is a promising approach against resistant pathogens [3]. aPDT relies on the interaction of a photosensitizer (PS) molecule, light, and molecular oxygen to generate reactive oxygen species (ROS), which induce targeted destruction of microorganisms [4]. Unlike conventional antibiotics, aPDT does not depend on a single molecular target, and the short interval between PS administration and irradiation further limits adaptive resistance mechanisms [3, 4].

Porphyrins have been widely used in aPDT against both Gram‐positive and Gram‐negative bacteria, showing significant bacterial growth inhibition at low concentrations [5]. The effectiveness of neutral porphyrin‐based aPDT is largely influenced by the outer membrane, mainly due to the complex cell envelope composed of a lipid bilayer, peptidoglycan layer, and lipopolysaccharides, which restricts PS uptake and limits photodynamic efficacy [6, 7, 8].

Beyond bacterial envelope permeability, the performance of porphyrin‐based PSs is also strongly influenced by physicochemical factors such as lipophilicity, aggregation behavior in aqueous media, acid–base properties, and photostability [9]. Excessive hydrophobicity often promotes self‐aggregation, which reduces fluorescence, singlet oxygen generation, and overall photodynamic efficiency [10, 11]. Therefore, achieving a balanced amphiphilicity that favors cellular interaction without inducing aggregation is a key challenge in PS design.

Capsaicinoids are naturally occurring phenolic compounds that have attracted interest in medicinal chemistry due to their broad range of biological activities and structural versatility [12]. The incorporation of capsaicinoid moieties into PSs may modulate overall physicochemical properties and interactions with biological environments, which are important factors influencing photodynamic performance. Therefore, the functionalization of PpIX with capsaicinoid fragments represents a promising strategy for investigating structure–property relationships in aPDT.

In this study, two novel PpIX derivatives functionalized with one or two capsaicinoid fragments (named PpIX‐capsaicin **1** and PpIX‐capsaicin **2**) were synthesized and evaluated as PSs for aPDT. The impact of mono‐ and difunctionalization on photophysical properties, singlet oxygen generation, aggregation behavior, acid–base properties, lipophilicity, photostability, and antimicrobial photodynamic efficacy was systematically investigated. The photoinactivation potential of the new compounds was assessed against both drug‐sensitive and MDR strains of *Staphylococcus aureus* and *Escherichia coli*, aiming to establish structure–property–activity relationships relevant to the rational design of porphyrin‐based PSs for antimicrobial applications.

## 2 Results and Discussion

### 2.1 Synthesis and Characterization

**SCHEME 1:**
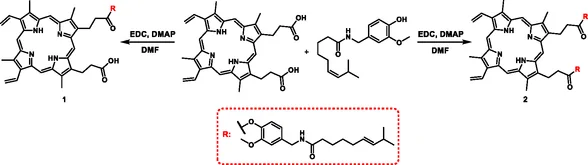
Synthesis of compounds 1 and 2. Reagents and conditions: (1) EDC, DMAP, DMF, rt, 72h, 17%. (2) EDC, DMAP, DMF, rt, 96h, 40%.

PpIX‐capsaicin **1** and **2** (Scheme 1) were designed to investigate the impact of mono‐ and dicapsaicinoid functionalization on the physicochemical, photophysical, and antimicrobial photodynamic properties of Protoporphyrin IX. Capsaicinoid moieties were selected due to their structural versatility and potential to modulate properties relevant to aPDT, including lipophilicity and interactions with biological environments [12]. Therefore, the conjugation of capsaicinoid fragments to PpIX was explored as a strategy to establish structure–property relationships and evaluate how the degree of functionalization influences photodynamic performance in bacteria.

Thus, both PpIX‐capsaicin derivatives were synthesized via Steglich esterification (Scheme 1), using 1‐ethyl‐3‐(3‐dimethylaminopropyl) carbodiimide hydrochloride (EDC) as the coupling agent and 4‐(dimethylamino)pyridine (DMAP) as the base in dimethylformamide (DMF). PpIX‐capsaicin **1** and PpIX‐capsaicin **2** were obtained in 17% and 40% yields, respectively. Both products were isolated as mixtures of ester derivatives due to the presence of dihydrocapsaicin (32%) in the commercial capsaicinoid mixture used for the synthesis. Dihydrocapsaicin is a naturally occurring capsaicinoid structurally related to capsaicin and commonly found as a major component in capsaicinoid extracts. For simplicity, the designations PpIX‐capsaicin **1** and PpIX‐capsaicin **2** are used throughout the manuscript, although both products comprise capsaicin‐ and dihydrocapsaicin‐derived esters.

^1^H NMR spectra of products **1** and **2** (Figures S1 and S4) show overall similar chemical shift patterns, reflecting the presence of the same porphyrinic core and capsaicinoid fragments. In product **2**, the observation of singlets corresponding to the twelve methyl protons attached to the porphyrin ring indicates a high degree of molecular symmetry and is consistent with diesterification. In contrast, product **1** displays a loss of this symmetry, evidenced by the splitting of the methyl signals into multiple singlets. In both products, methylene protons from the linker chains appear as triplets, and aromatic and aliphatic signals attributable to capsaicinoids are present, indicating that both capsaicin and dihydrocapsaicin fragments were incorporated.

The ^13^C NMR (APT) (Figures S2 and S5) spectra of both products show signals associated with ester and amide carbonyl carbons, as well as porphyrinic, aromatic, and aliphatic carbons. However, the coexistence of capsaicin and dihydrocapsaicin leads to significant signal overlap in both ^1^H and ^13^C NMR spectra, limiting detailed structural assignments based solely on nuclear magnetic resonance (NMR) data. Consequently, NMR analysis was combined with literature data (Table S2) and MS results (Figures S3 and S6), which were essential to confirm product formation and to establish that product **2** consists of a mixture of diesters, while product **1** corresponds to an asymmetrically substituted porphyrin derivative.

### 2.2 Quantum Yields of Fluorescence and (Φ_F_) Singlet Oxygen (Φ_Δ_)

Understanding photophysical parameters such as fluorescence and singlet oxygen quantum yields is essential for evaluating the applicability of new PSs in PDT [13]. The fluorescence quantum yields (Φ_F_) of PpIX‐capsaicin **1** and **2** were similar and remained below 0.1 (Table 1), consistent with the behavior typically observed for porphyrinic PSs, where intersystem crossing efficiently competes with fluorescence emission [15, 16]. Since fluorescence efficiency can be influenced by factors such as the molecular environment and flexibility, which affect nonradiative deactivation [17, 18], the slightly higher Φ_F_ values observed for both derivatives compared to PpIX suggest that capsaicinoid functionalization induces modest changes in the excited‐state deactivation processes. Although the increase was limited, the results indicate that structural modification of the porphyrin framework influences the photophysical properties of the system.

**TABLE 1:** Quantum yields of fluorescence (Φ_F_) and singlet oxygen (Φ_Δ_) determined for PpIX, PpIX‐capsaicin **1**, and PpIX‐capsaicin **2** in ethanol.

PSs	*Φ*_F_, *λ*_exc_ = 408 nm	*Φ*_Δ_, *λ*_exc_ = 400 nm	*τ*_Δ_, μs
PpIX	0.070^a^	0.92^a^	14.9^a^
			14.6
1	0.076	0.70 ± 0.03	14.4
2	0.081	0.80 ± 0.10	14.6

a Values extracted from [14].

The ^1^O_2_ quantum yield (Φ_Δ_) is a key parameter that assesses the efficiency of a PS in converting absorbed light energy into ^1^O_2_ in the presence of molecular oxygen. This value represents the fraction of absorbed photons that lead to the formation of ^1^O_2_ and ranges from 0 (no ^1^O_2_ generation) to 1 (100% efficiency). In photodynamic action, ^1^O_2_ is considered the main cytotoxic agent [19]. Due to its high reactivity, ^1^O_2_ initiates oxidative reactions in its microenvironment, targeting bacterial cell walls, lipid membranes, enzymes, and nucleic acids [19].

^1^O_2_ decay profile illustrates how the phosphorescence signal intensity at 1270 nm decreases over time following its formation. This curve reflects the deactivation kinetics of ^1^O_2_, which in homogeneous systems follow first‐order kinetics, indicating minimal interaction with other components [20, 21]. The initial phosphorescence intensity at 1270 nm is directly related to the efficiency of the PS in generating ^1^O_2_. A higher initial signal suggests more efficient energy transfer from the excited triplet state to molecular oxygen [21]. For the synthesized derivatives **1** and **2**, a slightly weaker initial emission was observed compared to the PpIX precursor (Figure S8), suggesting enhanced ^1^O_2_ generation efficiency upon incorporation of the capsaicin fragment.

The ^1^O_2_ lifetime was evaluated in ethanolic solution based on the recorded decay spectra (Figure S8). In this medium, *τ*_Δ_ is mainly influenced by solvent‐induced deactivation mechanisms involving electronic and vibrational energy transfers [14]. When no reaction occurs between the ^1^O_2_ and the PS, *τ*_Δ_ values can be compared to the standard (Table 1). The singlet oxygen quantum yield (Φ_Δ_) was calculated according to Equation (2) (Experimental Section).

Φ_Δ_ measurements were performed in ethanol, which was selected as a common solvent to ensure complete solubilization of all compounds and minimize aggregation effects, thereby enabling a direct comparison of their intrinsic photosensitizing efficiencies. Due to the different solubility of PpIX, PpIX‐capsaicin **1**, and PpIX‐capsaicin **2** in aqueous media, measurements in water or D_2_O would not provide equivalent experimental conditions for comparative analysis. Although the ΦΔ values (Table 1) show that PpIX‐capsaicin derivatives **1** and **2** generate less ^1^O_2_ than PpIX, their quantum yields fall within the range typically observed for many porphyrinic PSs [22], and therefore do not compromise their applicability in aPDT, since Type I photochemical reactions are also possible during the photosensitizing process and can be as damaging to biomolecules as ^1^O_2_.

### 2.3 UV–visible Absorption and Solvent‐Dependent Stability of PpIX‐Capsaicin Derivatives

**FIGURE 1:**
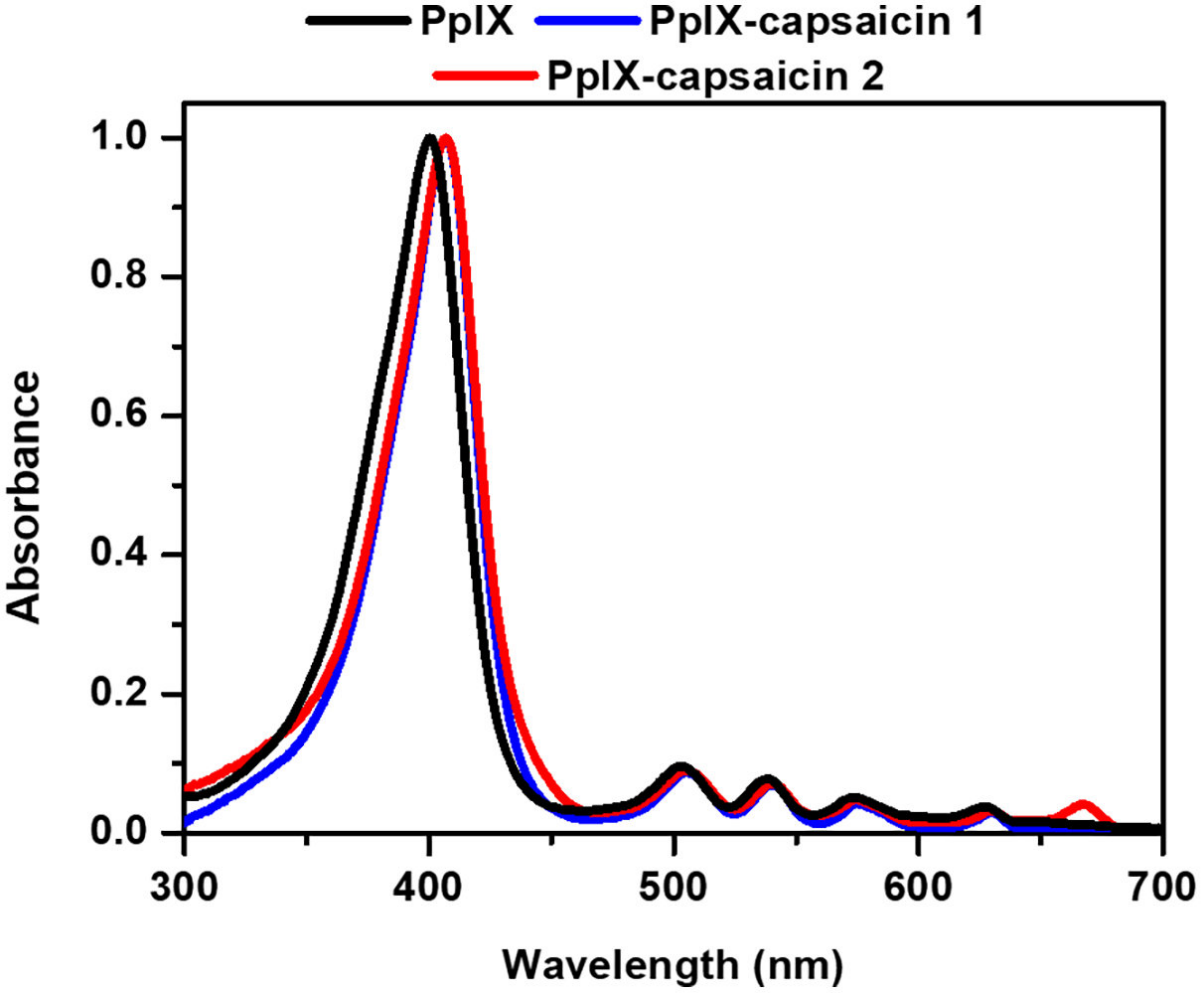
Spectrum of normalized absorption in the UV–visible in DMSO.

As shown in Figure 1, both PSs (**1** and **2**) exhibit Soret and Q bands absorption maxima situated at slightly higher wavelengths compared to PpIX in DMSO. These absorption bands are associated with π–π* electronic transitions between the HOMO and LUMO orbitals within the pyrrolic rings and are typical of base‐free porphyrins in their monomeric state [13, 23, 24]. Additionally, an absorption band at 668 nm was detected in the spectra of PpIX‐capsaicin **2**.

The absorption band observed at 668 nm led to the formulation of hypotheses based on spectral changes detected during the handling of compounds **1** and **2**. Upon solubilization in chloroform, PpIX‐capsaicin **2** underwent significant spectral modifications, including a bathochromic shift accompanied by an increase in Soret band intensity, the disappearance of the Q bands, and the appearance of two absorption bands between 500 and 600 nm. A similar spectral profile was also observed for PpIX‐capsaicin **1**. However, in this case, the changes developed with slower kinetics. In parallel, solutions exhibiting this spectral behavior showed a visible color change from red to green, suggesting a substantial alteration in the electronic structure of the porphyrinic core.

Another phenomenon observed was the gradual increase of a band around 668 nm for PpIX‐capsaicin **1** when solubilized in chloroform. In contrast to PpIX‐capsaicin **2**, this band was not initially present. For PpIX itself, no spectral changes were observed throughout the entire handling process. Taken together, these observations support two main hypotheses: (i) the establishment of an acid–base equilibrium between the porphyrin esters and acidic species present in chloroform, which could account for the spectral changes leading to the presence of only two Q bands [25]; and (ii) the occurrence of an accelerated photodegradation process, which may explain the emergence of the 668 nm band, already detected in derivative **2** since its isolation [26]. The only remaining uncertainty concerns the origin of the absorption band at 668 nm, whether it arises from the acid–base equilibrium itself or from a photodegradation process. Therefore, a p*K*_a_ assay was performed to investigate spectral changes during protonation.

### 2.4 Acid‐Base Properties and p*K*_a_ of PpIX‐Capsaicin 1

**FIGURE 2:**
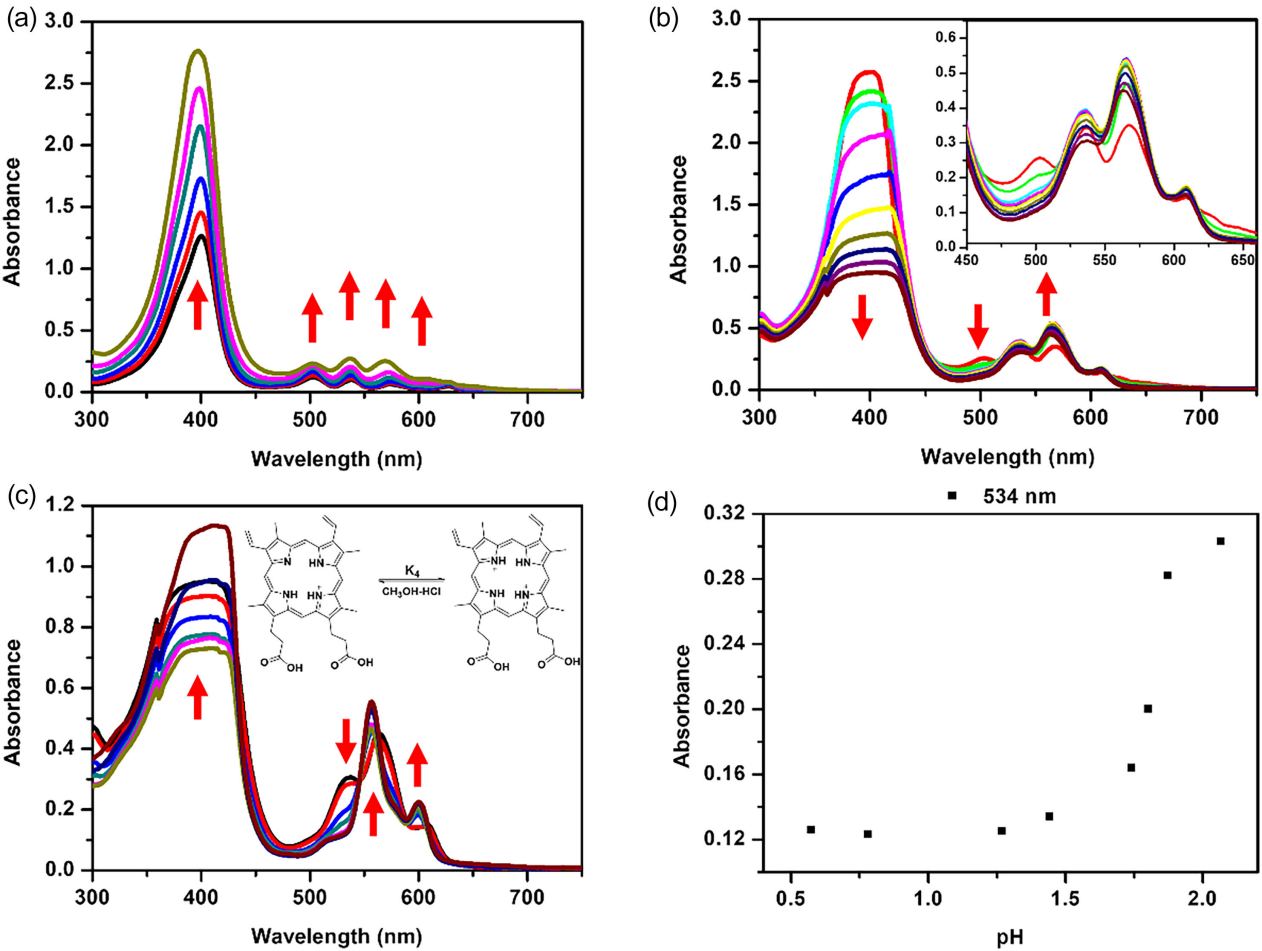
Spectrophotometric titration of PpIX at (a) pH = 4.69–4.15, (b) pH = 4.04–2.07, and (c,d) pH = 2.07–0.57, corresponding to the diprotonation equilibrium (*λ*_monoprotonated species_ = 534 nm), using HCl‐MeOH.

**FIGURE 3:**
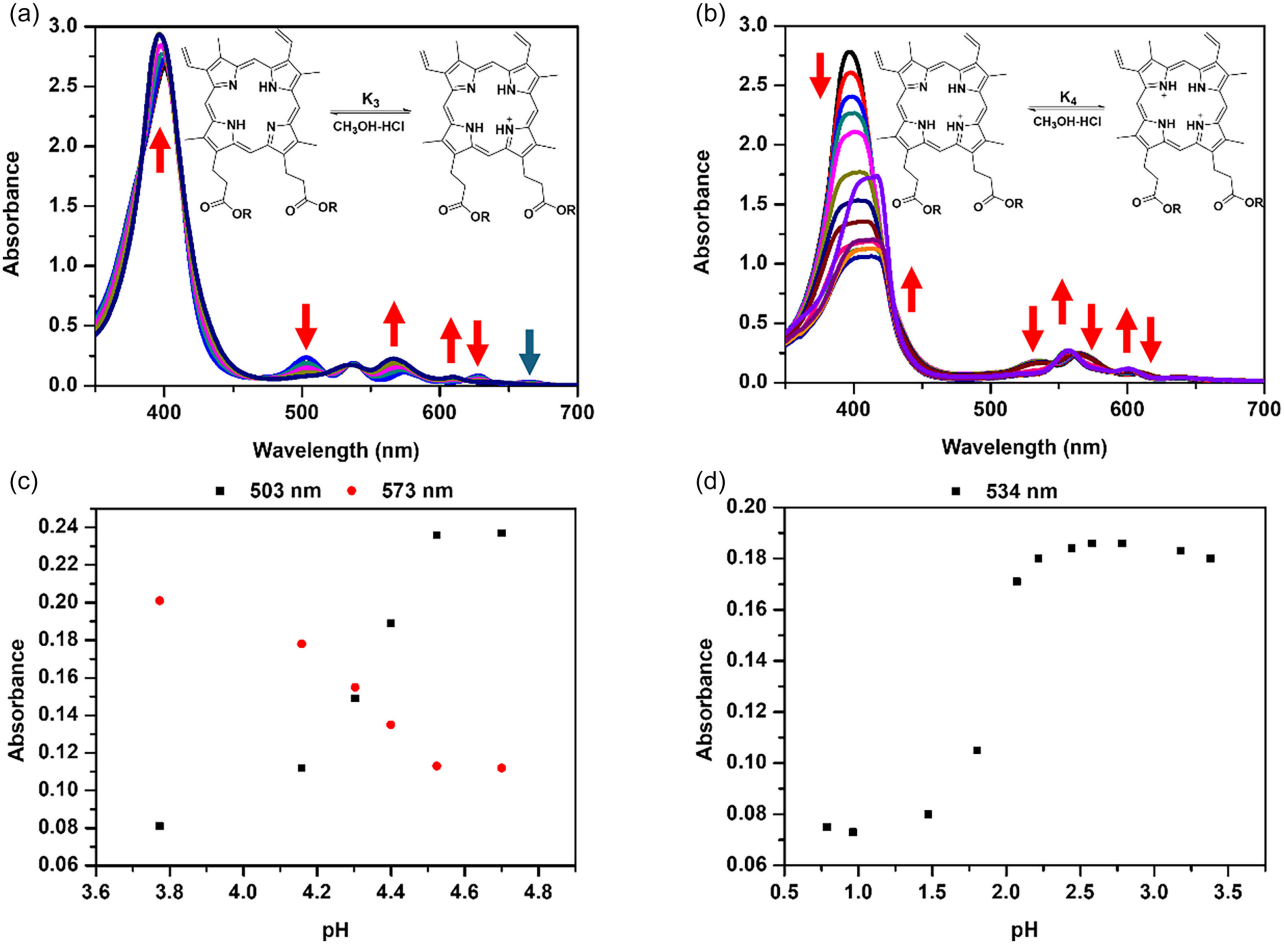
Spectrophotometric titration of PpIX‐capsaicin **1** at (a, c) pH = 4.69–3.77, corresponding to the monoprotonation equilibrium (*λ*_neutral species_ = 503 nm/*λ*_monoprotonated species_ = 573 nm), and at (b, d) pH = 2.22–0.57, corresponding to the diprotonation equilibrium (*λ*_monoprotonated species_ = 534 nm), using HCl‐MeOH.

Initially, the absorption band located at 668 nm (Figure 1) was hypothesized as a result from protonation equilibria, associated with the monoprotonation process, which has been previously observed in PpIX esters [25]. This assumption was based on the observation that the same band also appeared in PpIX‐capsaicin **1** when solubilized in chlorinated solvents for extended periods, conditions that can lead to protonation of pyrrolic rings through radical or acid–base mechanisms [27]. To investigate this, both PpIX and PpIX‐capsaicin **1** were titrated with HCl in anhydrous methanol over an apparent pH range from 4.69 to 0.17. Upon each addition of acid, the Soret band of PpIX progressively broadened (Figure 2). In contrast, the spectral response of PpIX‐capsaicin **1** to acidification differed significantly from PpIX (Figure 3).

For PpIX, an initial increase in the intensity of all absorption bands was observed during the first five additions of HCl‐methanol, corresponding to a pH range of 4.69–4.15 (Figure 2a). In the pH interval between 4.04 and 2.07 (Figure 2b), however, the expected monoprotonation process could not be reliably tracked due to the loss of isosbestic points, possibly due to aggregation behavior in this equilibrium [25]. Only the diprotonation process could be monitored (Figure 2c,d), as the isosbestic point at 545 nm persisted down to pH 0.57.

The acid–base equilibria of PpIX‐capsaicin **1** were more clearly delineated into two stages: monoprotonation (Figure 3a,c) and diprotonation (Figure 3b,d). During monoprotonation, the Soret band and the Q band at 574 nm increased in intensity and experienced a hypsochromic shift, while the bands at 503 and 628 nm decreased. Diprotonation, on the other hand, was characterized by a decrease followed by an increase and bathochromic shift of the Soret band, along with the emergence of new bands at 557 and 600 nm.

To determine the apparent p*K*_a_ values associated with each protonation step for PpIX and PpIX‐capsaicin **1**, specific wavelengths were selected that corresponded to distinct spectral changes, enabling the monitoring of interconversion between species as the medium became more acidic. A sigmoidal fit of absorbance versus pH data was used to determine these p*K*_a_ values, as presented in Table 2.

**TABLE 2:** Apparent p*K*_a_ values measured for PpIX, PpIX‐capsaicin 1, and PpIX‐capsaicin2.

PS	**p*K***_**3**_	**p*K***_**4**_
PpIX	Obscured by aggregation	+1.81
	a^a^	+2.37^a^
PpIX‐capsaicin **1**	+4.33	+1.89
PpIX‐capsaicin **2**	—	—
PpIX‐dimethyl ester^a^	+3.37^a^	+2.64^a^

a Extracted from [25].

The results revealed that the esterification of PpIX with a capsaicin fragment had a significant impact on its acid–base behavior, particularly in the monoprotonation equilibrium (p*K*_3_). When compared to PpIX‐dimethyl ester, the neutral form of PpIX‐capsaicin **1** exhibited increased basicity, despite the esterification of only one carboxylic acid group [25]. This suggests that the introduction of a second capsaicin fragment could further enhance the basicity of the pyrrolic core.

The experimental p*K*_4_ values for both PpIX and PpIX‐capsaicin **1** were relatively close, with a slight increase observed for the capsaicin derivative. However, the p*K*_4_ obtained for PpIX in this study was lower than the value reported by Hynninen (2014) [25], possibly due to a temperature‐induced shift in the acid–base equilibrium of the macrocycle [28]. Increased temperature promotes deprotonation of the pyrrolic core, thereby lowering the p*K*_a_. This discrepancy is likely due to the strict temperature control (20 °C) employed in Hynninen’s study, whereas temperature could not be regulated in the present work.

Comparative analyses between PpIX and PpIX‐capsaicin **1** revealed notable differences in their protonation‐induced spectral behaviors. PpIX exhibited a more complex spectral profile, making the identification of the monoprotonation equilibrium less straightforward. In contrast, PpIX‐capsaicin **1** displayed a distinct band at 668 nm, which may be indicative of degradation. These findings demonstrate that capsaicinoid functionalization alters the acid–base behavior and photochemical stability of the porphyrin.

### 2.5 Photobleaching

Photobleaching is defined as a process in which PS molecules irreversibly lose their ability to generate ROS after light exposure. This is driven by photochemical reactions that degrade the molecular structure, often initiated by interaction with ROS formed during photoexcitation [9]. To investigate the photodegradation process of porphyrin under different wavelengths, the compounds were irradiated using white or green LEDs. The aim was to assess the photostability of PpIX derivatives by analyzing UV–vis spectra over 90 min of irradiation, monitoring subtle variations in absorbance intensity, especially in compounds prone to rapid degradation. The spectra recorded over time were compiled (Figure S9), and the behavior of the Soret and Q bands of each porphyrin was evaluated upon irradiation time.

**FIGURE 4:**
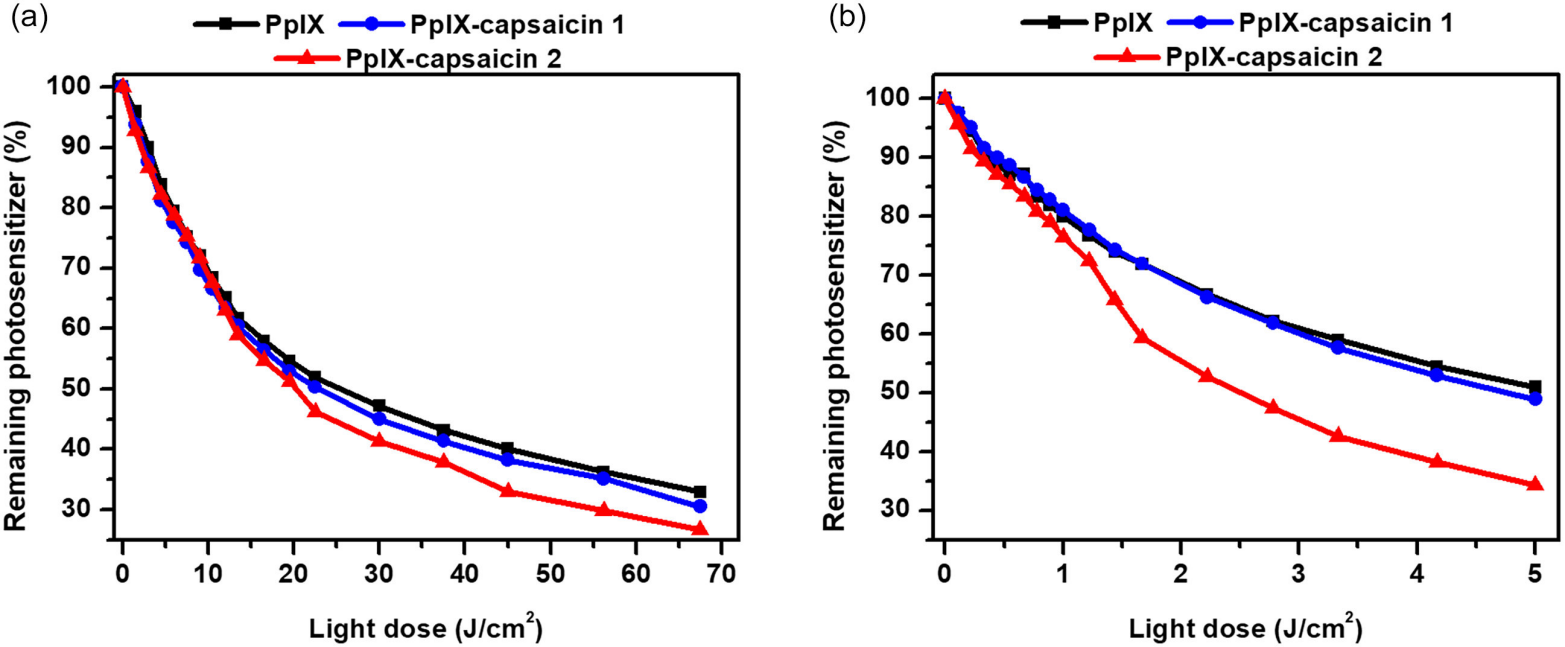
Remaining concentration (%) of each PS, PpIX (black line), PpIX‐capsaicin **1** (blue line), and PpIX‐capsaicin **2** (red line) under different irradiation setups: (a) white LED (0–70 J/cm^2^) and (b) green LED (0–5 J/cm^2^).

Under white LED irradiation (14.1 mW/cm^2^), PpIX was the least photodegraded compound, followed by PpIX‐capsaicin **1** and then PpIX‐capsaicin **2** (Figure 4a). This trend is likely related to differences in intrinsic photostability induced by structural modification, which may affect degradation pathways independently of ^1^O_2_ generation. Irradiation with the green LED (463.5 μW/cm^2^) was carried out for the same duration, but under lower light doses due to the lower power output of the LED. PpIX also was the least photodegraded compound, followed by PpIX‐capsaicin **1**, while PpIX‐capsaicin **2** exhibited a more intense photobleaching profile compared to the other porphyrins.

Analysis of degradation as a function of light dose (Figure 4) revealed that, at doses around 5 J/cm^2^, white LED irradiation led to ≈20% degradation in all PSs. In contrast, green LED irradiation resulted in 50% degradation for PpIX and PpIX‐capsaicin **1**, and over 60% for PpIX‐capsaicin **2**. At the maximum light dose (≈70 J/cm^2^), the white LED produced up to 60% photodegradation.

Upon irradiation, all absorption bands, including the Soret and the four Q bands, gradually decreased in intensity over time (Figure S9). However, a new band emerged in the 668–700 nm region, with its intensity increasing proportionally to the light dose. This behavior has also been reported by other authors and is typically associated with the formation of photoproducts that absorb in this spectral region [26]. Given that photodegradation is often linked to increased ROS generation, the presence of the 668 nm band in PpIX‐capsaicin **2**, even at the time of its isolation, suggests that this compound may have undergone partial photodegradation during the purification process. These results highlight the complexity of the photodegradation process and the importance of selecting an appropriate light source and PS for PDT applications.

### 2.6 Aggregation Behavior of PpIX‐Capsaicin 1 and PpIX‐Capsaicin 2 in Aqueous Solutions

PpIX has a large hydrophobic core and two hydrophilic side chains that tend to form aggregates in aqueous media. These aggregates result from intermolecular interactions between the COOH groups of the side chains and π–π stacking [29, 30]. During its application in PDT, low water solubility combined with aggregation compromises both fluorescence processes and the efficiency of ^1^O_2_ production [31].

**FIGURE 5:**
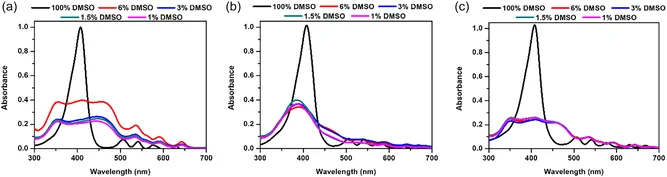
UV–visible absorption spectra of (a) PpIX, (b) PpIX‐capsaicin **1**, and (c) PpIX‐capsaicin **2**, varying proportions of water and DMSO.

The porphyrins were easily solubilized in 100% DMSO, showing UV–vis spectra with an intense Soret band and four less intense Q bands, characteristic of the monomeric forms of the compounds [29, 30]. As shown in Figure 5, when the DMSO content was reduced to 6%, all porphyrins displayed broadened and less intense Soret bands, which indicates aggregation.

For PpIX (Figure 5a) and PpIX‐capsaicin **2** (Figure 5c), the spectra showed Soret bands with three distinct regions, indicating the coexistence of monomeric species and both H‐type and J‐type aggregates in solution [29, 30]. H‐aggregates are associated with face‐to‐face stacking, while J‐aggregates arise from head‐to‐tail stacking. In contrast, PpIX‐capsaicin **1** (Figure 5b) at 6% DMSO showed a blue shift of the Soret band to 386 nm, indicating the predominance of H‐type aggregates [29, 30].

At 3% DMSO, PpIX showed the greatest decrease in absorbance intensity, in contrast, PpIX‐capsaicin **1** and **2** exhibited minimal changes in absorbance intensity and aggregate profile when the DMSO content was reduced from 6% to 1% (Figure 5). Regarding the Q bands, PpIX showed the most significant changes, including increased absorption intensity in the range 515–670 nm, red shift, and loss of spectral definition. PpIX‐capsaicin **2** maintained nearly unchanged absorption intensities, and PpIX‐capsaicin **1** showed a more distinct aggregate spectral profile, with a slight reduction in Q band absorption intensity.

Both derivatives exhibited greater spectral stability than PpIX upon reduction of DMSO content, particularly within the solvent range commonly employed in biological assays (1%–5% DMSO). However, distinct aggregation behaviors were observed between the two derivatives. PpIX‐capsaicin **1** displayed a simpler aggregation profile, predominantly characterized by H‐type aggregates, whereas PpIX‐capsaicin **2** maintained the coexistence of H‐ and J‐type aggregates across all solvent conditions evaluated. This persistence of aggregated species suggests that the higher hydrophobicity introduced by the second capsaicinoid unit favors intermolecular interactions rather than improved molecular dispersion. Therefore, although capsaicinoid functionalization altered the aggregation behavior of PpIX, increasing the degree of substitution did not further reduce aggregation and may instead stabilize aggregated species.

### 2.7 Partition Coefficient

The efficacy of these compounds depends on structural factors that influence their cellular uptake, such as the Log *P*, which reflects the physicochemical nature of the compound [9]. The balance between hydrophilic and hydrophobic moieties, along with the overall lipophilicity of the PS, plays a crucial role in the effectiveness of aPDT. The *n*‐octanol/water Log *P* is widely accepted as a predictor of the biological activity of drugs and other bioactive compounds [9].

**FIGURE 6:**
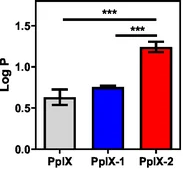
Bars graph of the Log *P* values shown in Table 2. The experiment was performed in triplicate (*n* = 3), and error bars represent standard deviation. ***p < 0.0001 indicates statistically significant differences according to ANOVA.

In this context, the Log *P* of each porphyrin was determined using the *n*‐octanol/water system via the shake‐flask method to evaluate changes in lipophilicity resulting from the addition of capsaicin fragments to PpIX. The results are presented in Table 3 and visually represented in the graph in Figure 6.

**TABLE 3:** Log *P* values measured for PpIX, PpIX‐capsaicin **1**, and PpIX‐capsaicin **2**.

PS	Log *P*
PpIX	0.63 ± 0.07
PpIX‐capsaicin **1**	0.75 ± 0.01
PpIX‐capsaicin **2**	1.24 ± 0.04

Based on the data obtained, the addition of a single capsaicinoid fragment slightly increased lipophilicity, whereas the addition of a second fragment resulted in an approximately two‐fold increase compared to PpIX. Therefore, modification of the PpIX chromophore significantly affected lipophilicity only after incorporation of the second capsaicinoid unit, as illustrated in Figure 6.

These results further demonstrate that increasing the degree of capsaicinoid functionalization does not lead to a proportional improvement in properties relevant to biological applications. While PpIX‐capsaicin **2** exhibited substantially higher lipophilicity, excessive hydrophobicity may limit its interaction with aqueous biological environments. In contrast, the moderate increase observed for PpIX‐capsaicin **1** suggests a more balanced hydrophilic–lipophilic profile, which is generally considered favorable for biological applications. Therefore, these findings indicate that an optimal range of lipophilicity, rather than maximal hydrophobicity, is more desirable for photodynamic applications.

### 2.8 PhotoDynamic Inactivation (PDI) of *S. aureus* and *E. coli*

aPDT is a promising approach to address the growing threat of MDR bacteria [3]. This technique is based on the photosensitization of pathogens by PSs and presents several advantages over traditional antibiotics. These include: (a) the PS is nontoxic in the absence of light, which prevents bacteria from sensing the need to develop resistance mechanisms against the compound; (b) the interval between PS administration and irradiation is short, reducing the chance of resistance development; and (c) once irradiated, the resulting cellular damage is rapid and severe enough to hinder genetic exchange mechanisms, thereby preventing resistance to aPDT from developing [3]. In this study, PpIX and its derivatives modified with capsaicinoid fragments (PpIX‐capsaicin **1** and **2**) were assayed for the inactivation of both Gram‐positive and Gram‐negative bacteria (*S. aureus* and *E. coli*), including both sensitive and MDR strains.

**FIGURE 7:**
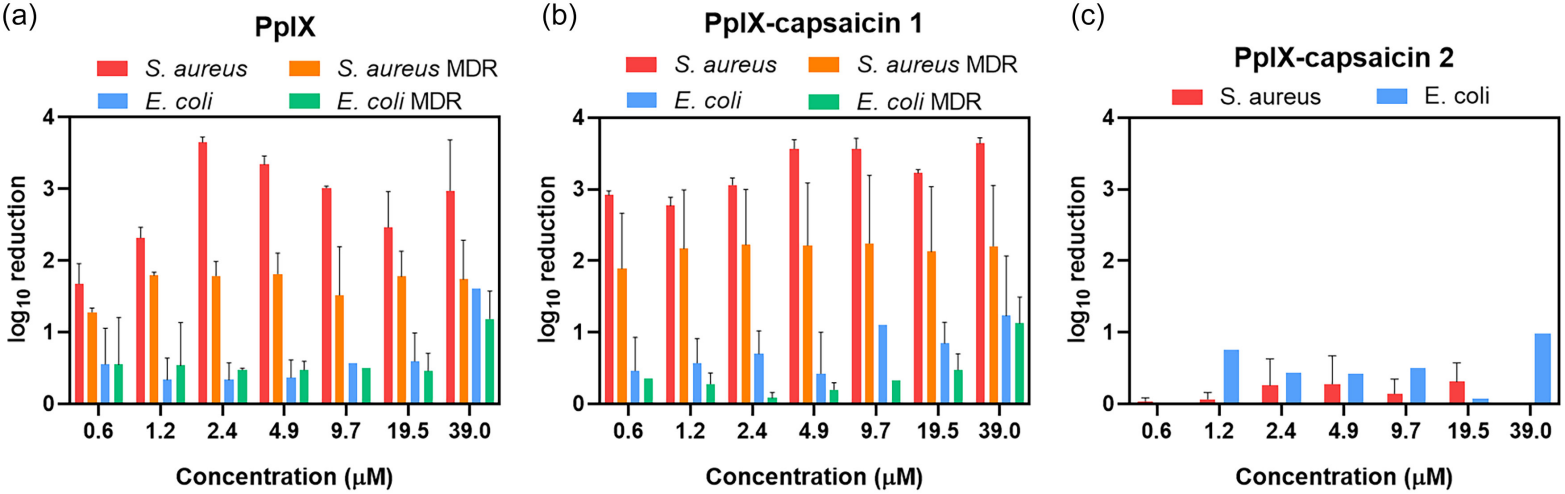
Reduction of *S. aureus* (ATCC 29213), *S. aureus* (ATCC BAA‐44), *E. coli* (ATCC 25922), and *E. coli* (ATCC BAA‐198) populations by aPDT as a function of (a) PpIX, (b) PpIX‐capsaicin **1**, and (c) PpIX‐capsaicin **2**concentrations. Each bar represents the mean ± standard deviation. After PS incubation, bacteria were kept at 37°C and protect from light (in the dark) or irradiated with white LED at 30 J/cm^2^.

For the drug‐sensitive strain of *S. aureus* (ATCC 29 213), all studied porphyrins showed no antibacterial activity in the absence of light. However, following irradiation, a significant bacterial inactivation was observed for PpIX (Figure 7a) and PpIX‐capsaicin **1** (Figure 7b), confirming their efficacy as a PS.

PpIX reduced bacterial viability by ~1.7 logs at the lowest concentration (0.6 µM), reaching a maximum reduction of ~3.3 logs at 1.2 µM (Figure 7a). This plateau in activity may be associated with self‐aggregation and photodegradation processes at higher PS concentrations [32].

PpIX‐capsaicin **1** (Figure 7b) also demonstrated significant photoinactivation, achieving a ~3‐log reduction at the lowest concentration (0.6 µM) and ~3.6 logs at 9.75 µM. According to commonly accepted microbiological criteria, reductions of ≥3‐log colony‐forming unit (CFU) are considered biologically relevant and indicative of bactericidal activity [33, 34]. In contrast, PpIX‐capsaicin **2** (Figure 7c) showed no significant reduction in viable cells, even after irradiation. This reduced activity was accompanied by pronounced self‐aggregation, increased photodegradation, and higher lipophilicity compared to PpIX‐capsaicin **1**, factors that may have negatively influenced its photodynamic performance. Due to the lack of phototoxicity observed for PpIX‐capsaicin **2** against the drug‐sensitive S. aureus strain, this compound was not further evaluated against the MDR S. aureus (ATCC BAA‐44) strain.

For the MDR *S. aureus* strain (ATCC BAA‐44), a similar trend was observed. PpIX (Figure 7a) reduced bacterial counts by ~1.28 logs at the lowest concentration (0.6 µM) and ~1.78 logs at 19.5 µM. PpIX‐capsaicin **1** (Figure 7b), however, showed greater efficiency, reducing ~1.89 logs at the lowest concentration and up to ~2.2 logs at 39 µM. These results indicate that PpIX‐capsaicin **1** was more effective than PpIX against both drug‐sensitive and MDR *S. aureus* strains. This enhanced activity was accompanied by a lower aggregation tendency while maintaining singlet oxygen generation comparable to that of PpIX.

In the case of drug‐sensitive *E. coli*, both PpIX (Figure 7a) and PpIX‐capsaicin **1** (Figure 7b) showed less pronounced photoinactivation compared to *S. aureus*. PpIX reduced viability by ~0.55 logs at 0.6 µM and ~1.12 logs at 39 µM. PpIX‐capsaicin **1** reached a maximum reduction of ~1.23 logs at the highest concentration, while PpIX‐capsaicin **2** showed no consistent pattern of photoinactivation. The lower effectiveness against *E. coli* is likely related to the limited permeability of the PS through the bacterial envelope, due to the double lipid bilayer structure characteristic of Gram‐negative bacteria [3].

Finally, the MDR *E. coli* strain (ATCC BAA‐198) displayed similar results to its sensitive counterpart. At the lowest concentration (0.6 µM), PpIX (Figure 7a) reduced viability by ~0.54 logs, while PpIX‐capsaicin **1** (Figure 7b) achieved a reduction of only ~0.12 logs at 0.6 µM. At 39 µM, both PSs led to similar reductions (~1.19 logs for PpIX and ~1.13 logs for PpIX‐capsaicin **1**).

Overall, the results highlight the potential of aPDT for bacterial photoinactivation and demonstrate that the biological performance of the evaluated compounds depends on a balance of physicochemical properties. PpIX‐capsaicin **1** exhibited superior activity against both drug‐sensitive and MDR *S. aureus* strains, whereas PpIX‐capsaicin **2** showed no significant photoinactivation despite its higher lipophilicity. The reduced activity of PpIX‐capsaicin **2** was accompanied by greater aggregation, lower photophysical performance, and increased hydrophobicity, suggesting that excessive lipophilicity may be detrimental to photodynamic efficacy. Limited photoinactivation was observed for *E. coli* strains, consistent with the permeability barriers associated with the Gram‐negative cell envelope [3]. Together, these findings demonstrate that increasing the degree of capsaicinoid functionalization does not necessarily improve antimicrobial photodynamic activity and reinforce the importance of balancing molecular properties in PS design.

## 3 Conclusion

This study investigated novel PpIX‐based PS for aPDT against Gram‐positive and Gram‐negative bacteria. Two capsaicin‐conjugated derivatives, PpIX‐capsaicin **1** and PpIX‐capsaicin **2**, were synthesized and evaluated for their spectroscopic properties, photostability, and antimicrobial activity against both sensitive and MDR strains.

Spectroscopic analyses revealed significant electronic changes in the derivatives, accompanied by increased photodegradation and enhanced pyrrolic basicity with increasing numbers of capsaicinoid units. Among the evaluated compounds, PpIX‐capsaicin **1** showed improved performance compared to PpIX, particularly in the photoinactivation of *S. aureus*. This enhanced activity was associated with its lower aggregation tendency and moderate increase in lipophilicity, properties that may favor interactions with bacterial cells while maintaining suitable photophysical performance. In contrast, PpIX‐capsaicin **2** showed no significant photoactivity, which was accompanied by persistent aggregation, increased photodegradation, and substantially higher lipophilicity.

Limited photoinactivation was observed for *E. coli*, likely reflecting the lower permeability of the Gram‐negative cell envelope. Overall, these findings demonstrate that increasing the degree of capsaicinoid functionalization does not result in a proportional enhancement of photodynamic activity. Instead, the addition of a second capsaicinoid unit was associated with increased aggregation, reduced photophysical performance, and excessive lipophilicity, factors that may compromise biological activity. These results highlight the importance of achieving an optimal balance between lipophilicity, solubility, and molecular organization, reinforcing that, in the design of porphyrin‐based PSs, more is not necessarily better.

## 4 Experimental Section

### 4.1 Reagents

Commercial reagents and solvents were used without further purification. PpIX, DMAP, and Capsaicin (60% pure) were purchased from Sigma–Aldrich, and EDC from Oakwood Chemical. DMF was dried with molecular sieves. Flash column chromatography was performed using Macherey–Nagel silica gel (230–400 mesh ASTM). Analytical thin‐layer chromatography (TLC) was performed on chromatography aluminum sheets impregnated with silica‐gel 60 F_254_ (Sigma–Aldrich) and plate revelation was achieved using UV light (254 nm) and/or iodine atmosphere.

### 4.2 Methods

^1^H and proton‐decoupled ^13^C APT NMR spectra were acquired in CDCl_3_ at 400 MHz (^1^H) and 101 MHz (^13^C) (Bruker Ascend 400). Chemical shifts (*δ*) are reported in parts per million (ppm) using residual nondeuterated solvent peak as an internal standard (CDCl_3_: 7.26 ppm, TMS: 0.00 ppm for ^1^H NMR spectra; and CDCl_3_: 77.16 ppm for ^13^C NMR spectra), (acetone‐d_6_ 2.05 ppm, TMS: 0.00 ppm for ^1^H NMR spectra; and acetone‐d_6_: 29.84 ppm for ^13^C NMR spectra). Peak multiplicity was reported using the following abbreviations: s = singlet, d = doublet, t = triplet, q = quartet, dd = doublet of doublets, m = multiplet. The multiplicity is followed by the coupling constant(s) in Hz and integration. The values of the coupling constants were measured using MestReNova. For ^13^C APT NMR spectra, peaks pointing upwards correspond to methine (CH) and methyl (CH_3_) carbons, and peaks pointing downwards correspond to nonhydrogenated and methylene (CH_2_) carbons. High‐resolution mass spectrometry (HRMS) spectra were acquired using electrospray ionization (ESI) on a Q‐TOF instrument (Impact II, Bruker Daltonics, Bremen, Germany), operating in positive ion mode. Prior to analysis, the samples were resuspended in 500 μL of MeOH and injected into the mass spectrometer. Spectra were acquired at 1.00 Hz over a mass range of 50–1400 *m*/*z*. Analyses were performed under the following parameters: capillary voltage of 4.0 kV; source temperature of 180 °C; and dry gas flow of 4 L min^− 1^. A syringe pump (KDS Legato 100, KD Scientific, Holliston, MA, USA) was used at a flow rate of 10 µL min^−1^ to infuse the calibrant solution of sodium formate (10 mmol L^−1^; isopropanol:water; 1:1 v:v).

UV–vis absorption spectra were recorded using a Shimadzu UV‐2501 PC spectrophotometer and a Cary 5000 Agilent spectrophotometer operating in a range of 350–800 nm. Fluorescence emission spectra were measured using a Horiba FluoroMax‐4 fluorescence spectrophotometer, using a quartz cell with 1.0 cm optical with excitation and emission slit widths of 1 and 5 nm, respectively. Singlet oxygen phosphorescence values were determined from phosphorescence decay curves at 1270 nm. The data were recorded with a time‐resolved NIR fluorometer (Edinburgh Analytical Instruments) equipped with a Nd:YAG laser (Continuum Surelite III) for sample excitation at 400 nm. The emitted light was passed through a silicon filter and a monochromator before detection by NIR‐PMT (Hamamatsu Co. R5509).

### 4.3 Synthesis and Characterization

#### 4.3.1 Synthesis of PpIX‐Capsaicin **1**

PpIX (0,053 mmol) was solubilized in DMF (3 mL) and DMAP (0,005 mmol), and Capsaicin (0,053 mmol) were added to the reaction mixture. A solution of EDC (0.053 mmol) in DMF (2 mL) was then added dropwise over 24 h at room temperature. The reaction mixture was stirred at room temperature for 72 h in the dark and monitored by TLC. After completion, distilled water (10 mL) was added, and the solution was extracted with DCM (3 × 10 mL). The combined organic layers were dried over anhydrous Na_2_SO_4_, filtered, and concentrated under reduced pressure. The residue was purified by flash column chromatography using a mix of DCM/MeOH (98:2) to yield the PpIX‐capsaicin **1**.

PpIX‐capsaicin **1**: dark purple solid, 7.8 mg, 17%. ^1^H NMR (400 MHz, acetone‐*d*_6_) *δ* 10.11 (s, 1H), 10.06 (d, *J* = 8.3 Hz, 2H), 10.00 (s, 1H), 8.33 (dd, *J* = 18.0, 12.0 Hz, 2H), 7.34 (s, 1H), 6.87 (s, 1H), 6.66 (q, *J* = 7.7 Hz, 3H), 6.37 (d, *J* = 17.2 Hz, 2H), 6.16 (d, *J* = 11.2 Hz, 3H), 5.31 (t, *J* = 5.2 Hz, 2H), 4.44 (s, 3H), 4.30 (s, 3H), 4.26 (s, 3H), 3.66–3.60 (m, 8H), 3.58 (s, 8H), 3.52 (d, *J* = 7.2 Hz, 4H), 3.37 (s, 4H), 3.18 (s, 3H), 2.52 (s, 5H), 2.16 (t, *J* = 7.2 Hz, 4H), 1.61–1.52 (m, 4H), 1.29 (d, *J* = 10.2 Hz, 5H), 0.89 (d, *J* = 6.8 Hz, 6H), 0.81 (d, *J* = 6.4 Hz, 4H).^13^C NMR (101 MHz, Acetone‐*d*_6_) *δ* 206.26, 174.49, 172.02, 140.03, 139.70, 138.74, 131.48, 127.82, 123.55, 121.53, 120.35, 112.75, 98.93, 98.41, 98.15, 56.23, 43.42, 43.32, 37.41, 37.36, 33.08, 31.84, 28.09, 26.73, 26.24, 23.26, 22.58, 13.08, 12.01, 11.89. HRMS (ESI^+^): calcd for C_52_H_60_N_5_O_6_^+^ [M + H]^+^ = 850.4538 *m*/*z*, found [M + H]^+^ = 850.4538 *m*/*z*, error = 0.00 ppm.

#### 4.3.2 Synthesis of PpIX‐Capsaicin 2

PpIX (0,053 mmol) was solubilized in DMF (1 mL) and EDC (0,1 mmol), DMAP (0,005 mmol), and Capsaicin (0,079 mmol) were added to the reaction mixture. The reaction was stirred at room temperature in a light‐protected environment for 96h and was monitored by TLC. After completion, distilled water (10 mL) was added, and the solution was extracted with DCM (3 × 10 mL). The combined organic layers were dried over anhydrous Na_2_SO_4_, filtered, and concentrated under reduced pressure. The residue was purified by flash column chromatography using a mix of DCM/MeOH (98:2) to yield the PpIX‐capsaicin **2**.

PpIX‐capsaicin **2**: dark purple solid, 24.3 mg, 40%. ^1^H NMR (400 MHz, CDCl_3_) *δ* 10.16 (s, 1H), 10.12 (s, 1H), 10.09 (s, 1H), 10.02 (s, 1H), 8.28 (dd, *J* = 11.5, 1.5 Hz, 1H), 8.24 (dd, *J* = 11.4, 1.6 Hz, 1H), 6.69–6.61 (m, 4H), 6.58 (d, *J* = 8.1 Hz, 2H), 6.37 (d, *J* = 18.6 Hz, 2H), 6.19 (d, *J* = 11.5 Hz, 2H), 5.91 (d, *J* = 4.7 Hz, 2H), 5.33 (dd, *J* = 8.7, 5.7 Hz, 3H), 4.46 (t, *J* = 7.6 Hz, 4H), 4.23 (d, *J* = 5.6 Hz, 4H), 3.69 (d, *J* = 3.3 Hz, 6H), 3.62 (d, *J* = 1.5 Hz, 6H), 3.47 (t, *J* = 7.5 Hz, 5H), 3.32 (d, *J* = 1.7 Hz, 6H), 2.12 (t, *J* = 7.6 Hz, 4H), 1.96 (dd, *J* = 13.8, 6.9 Hz, 3H), 1.56 (s, 8H), 1.30–1.20 (m, 7H), 0.94 (d, *J* = 6.7 Hz, 8H), 0.85 (d, *J* = 6.6 Hz, 5H). ^13^C NMR (101 MHz, CDCl_3_) *δ* 173.07, 171.48, 151.21, 139.20, 138.23, 137.39, 130.45, 128.73, 128.37, 127.73, 126.63, 122.92, 121.04, 119.94, 112.09, 98.23, 97.59, 97.26, 96.63, 77.16, 55.65, 43.45, 39.12, 36.82, 36.65, 32.37, 31.10, 29.79, 29.54, 29.47, 28.09, 27.39, 25.93, 25.41, 22.79, 22.77, 21.94, 12.85, 11.91. HRMS (ESI^+^): calcd for C_70_H_85_N_6_O_8_^+^ [M + H]^+^ = 1137.6423 *m*/*z*, found [M + H]^+^ = 1137.6375 *m*/*z*, error = −4.22 ppm.

### 4.4 Photophysical Parameters

#### 4.4.1 Determination of Fluorescence Quantum Yield

Fluorescence quantum yield (Φ_F_) was determined by comparing the integrated areas under the emission spectra with that of a standard, following established procedures [14]. PpIX in ethanol was used as the reference (*λ*_exc_ at 408 nm, *λ*_em_ = 550–800 nm, and Φ_F_ = 0.08) [14, 15]. The absorbance at the excitation wavelength (Soret band) was adjusted to < 0.30 for all samples (PpIX, PpIX‐capsaicin **1**, and PpIX‐capsaicin **2**) to minimize inner filter effects and recorded in ethanol. Φ_F_ values were calculated using Equation (1) [35].

(1) $$\Phi_{F}=\Phi_{R}\frac{I}{I_{R}}\frac{A_{R}}{A}$$

where the subscript *R* refers to PpIX, Φ_F_ is the fluorescence quantum yield, *I* is the integral of the fluorescence band, and *A* is the absorbance at the excitation wavelength.

#### 4.4.2 *Determination of Singlet Oxygen* (^1^ *O*_2_) *Quantum Yield*

Singlet oxygen emits phosphorescence in the near‐infrared region (*λ*_max_ ≈ 1270 nm). Therefore, the phosphorescence decay at 1270 nm of PpIX‐capsaicin **1** and PpIX‐capsaicin **2** in ethanol at room temperature was recorded, using PpIX as the reference standard (*λ*_exc_ at 400 nm and Φ_Δ_ = 0.92) [14]. The singlet oxygen quantum yield (Φ_Δ_) was calculated using Equation (2).

(2) $$\Phi_{\text{Δ}}^{\text{sample}}=\Phi_{\text{Δ}}^{\text{reference}}\frac{I_{\text{Δ}}^{\text{sample}}}{I_{\text{Δ}}^{\text{reference}}}\frac{A_{\text{reference}}}{A_{\text{sample}}}$$

where Φ_Δ_^reference^ is the singlet oxygen quantum yield of the reference (PpIX), *I*_Δ_ is the phosphorescence intensity of singlet oxygen at 1270 nm, and *A* is the absorbance of the PSs [36].

### 4.5 Aggregation Behavior of PpIX‐Capsaicin 1 and PpIX‐Capsaicin 2 in Aqueous Solutions

PpIX, PpIX‐capsaicin **1**, and PpIX‐capsaicin **2** solutions were prepared by diluting aliquots of a concentrated stock solution in DMSO. The aliquot volume was adjusted so that the final 2 mL solution (100% DMSO) had an absorbance of ≈1.0 at 408 nm. Subsequently, solutions with DMSO concentrations of 6%, 3%, 1.5%, and 1% (v/v in water) were prepared. Absorption spectra for each solution were recorded using a Shimadzu UV‐2501 PC spectrophotometer. Decreases in the Soret band and other spectral variations were analyzed as indicators of self‐aggregation [9].

### 4.6 Partition Coefficient

The partition coefficient (Log *P*) was determined using the shake‐flask method. Due to the partial solubility of porphyrins in the *n*‐octanol/water system, 0.3 mg of each compound was dissolved in 100 μL of DMSO and vortexed until fully dissolved. Then, water and *n*‐octanol in the same proportions were added to the flask. The mixture was vortexed for 10 min and then allowed to stand to ensure proper phase separation. Subsequently, 200 μL from each phase was diluted in of DMSO for absorbance measurement using a spectrophotometer. The Log *P* value was calculated using Equation (3) [9, 37].

(3) $$\mathrm{Log}\;P=\mathrm{Log}\frac{A_{n\text{‐octanol}}}{A_{\text{water}}}$$

where the subscript Log *P* refers to the partition coefficient and *A* represents the absorbance of each phase at the absorption maxima of PpIX, PpIX‐capsaicin **1**, and PpIX‐capsaicin **2**.

### 4.7 p*K*_a_

#### 4.7.1 HCl‐MeOH Solution Preparation

The HCl/methanol solution was prepared under an argon atmosphere by the dropwise addition of 17 mL of 37% hydrochloric acid, using a separatory funnel, into 12 mL of concentrated sulfuric acid placed in a sealed filtering flask. The generated HCl gas was bubbled into a 50 mL flask containing 25 mL of methanol, cooled in an ice bath and stirred, via a glass tube connected to the setup. An aliquot of the final solution (2.5 mL) was diluted to 10 mL and titrated in triplicate using 0.100 g of calcium carbonate dissolved in 50 mL of distilled water, with 0.01% (w/w) methyl orange as indicator. The final concentration of the HCl/methanol solution was determined to be 2.5 mol L^−1^.

#### 4.7.2 Spectrophotometric Titration of the Porphyrins

For the titration of the porphyrins, new solutions were prepared by diluting the 2.5 mol L^−1^ HCl‐MeOH stock solution to 0.01, 0.1, and 1 mol L^−1^. Aliquots of each dilution were added to PpIX and PpIX‐capsaicin **1** in methanol, according to the procedure described in [25]. After each addition (Table S1), absorption spectra were recorded using a Shimadzu UV‐2501 PC spectrophotometer.

#### 4.7.3 Photobleaching

Porphyrin solutions were prepared by diluting a concentrated stock solution in DMSO. The diluted samples were adjusted to an absorbance of ≈1.0 at 408 nm (Abs408 nm ≈ 1) and irradiated at room temperature using either a green LED (463.5 μW/cm^2^, 15 cm distance) or a white LED (14.1 mW/cm^2^, 10 cm distance). Absorption spectra were recorded using a Shimadzu UV‐2501 PC spectrophotometer after cumulative irradiation periods of 2, 4, 10, 15, 30, 45, 60, 75, and 90 min. Irradiation was paused only during spectral acquisition and immediately resumed thereafter. The corresponding accumulated light doses ranged from 0 to 5 J/cm^2^ for the green LED and from 0 to 70 J/cm^2^ for the white LED. Photodegradation was evaluated by monitoring the decrease in the intensity of the Soret band [9].

#### 4.7.4 PDI of *S. aureus* and *E. coli*

The antibacterial activity was assessed against *S. aureus* (ATCC 29213) and *E. coli* (ATCC 25 922), both susceptible to most antimicrobials, as well as against MDR strains *S. aureus* (ATCC BAA 44) and *E. coli* (ATCC BAA 198), which are resistant to more than three antimicrobial classes. All bacterial strains were obtained from the American Type Culture Collection (ATCC) and cryopreserved at –80 °C.

Stock solutions of PpIX, PpIX‐capsaicin **1**, and PpIX‐capsaicin **2** were prepared by dissolving 1 mg of each PS in DMSO. These were further diluted in Brain Heart Infusion (BHI) broth to prepare working solutions. Serial dilutions were performed in 96‐well microplates using the standard broth microdilution method to obtain final concentrations ranging from 0.6 to 39 µM [9, 38, 39].

Bacterial suspensions were adjusted to a 0.5 McFarland standard (1.5 × 10^8^ CFU/mL). Plates were incubated in the dark at 37 °C for 15 min to allow PS uptake by the bacterial cells [38, 40]. Following incubation, one plate was kept in the dark (control), or irradiated with white LED light at a dose of 30 J/cm^2^.

Immediately after irradiation, bacterial suspensions were serially diluted (10^−1^ to 10^−7^) in BHI [40]. Aliquots from each dilution were plated in duplicate on Mueller Hinton Agar and incubated at 37 °C for 24 h for the Gram‐positive strains and 16 h for the Gram‐negative strains, followed by CFU counting [40, 41].

## Funding

This work was supported by the Fundação de Amparo à Pesquisa do Estado de Minas Gerais (Grants APQ‐02393‐24, APQ‐00864‐25), Conselho Nacional de Desenvolvimento Científico e Tecnológico (Grant 407282/2023‐8, 308094/2025‐5) and Coordenação de Aperfeiçoamento de Pessoal de Nível Superior (Grants 001, PROEX, 88887.131921/2025‐00).

## Conflicts of Interest

The authors declare no conflicts of interest.

## Supporting information

Supplementary Material

## Data Availability

The data that support the findings of this study are available from the corresponding author upon reasonable request.
