# Supplementary material for: Effects of Mono‐ and Dicapsaicinoid Functionalization on the Physicochemical Properties and Antimicrobial Photodynamic Activity of Protoporphyrin IX
Source: ChemMedChem. 2026 Sep 7;21(17):e70475. doi: 10.1002/cmdc.70475 (PMC13549795; doi:10.1002/cmdc.70475)
Supplement: Supplementary file 1 [file CMDC-21-e70475-s001.pdf]

Supporting Information

Effects of Mono- and Di-Capsaicinoid Functionalization on the Physicochemical Properties and Antimicrobial Photodynamic Activity of Protoporphyrin IX

Ieda Vieira da Cunha[a], Gabriel Guimarães Calefi[b], Nagela Bernadelli Sousa Silva[b], Júlia Gomes Teixeira[b], Marcos Eduardo Gomes do Carmo[a], Paula Derksen Macruz[c], Eduardo Jorge Pilau[c], Antônio Otávio de Toledo Patrocínio[a], Carlos Henrique Gomes Martins[b], Celso de Oliveira Rezende Júnior[a], [d] and Tayana Mazin Tsubone*[a]

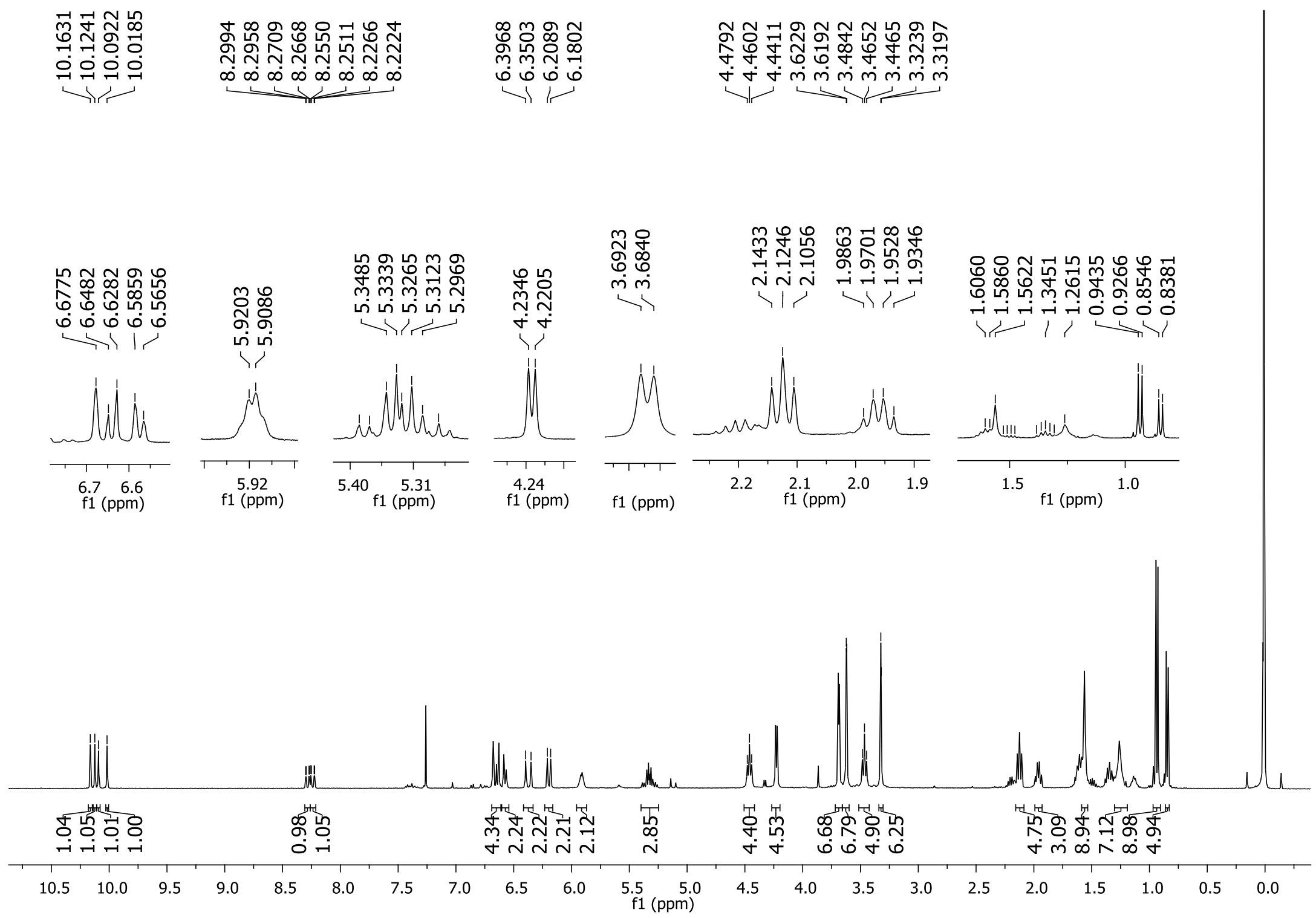

Figure S1. ^{1}H NMR spectrum (400 MHz, $CDCl_3$) of PpIX-capsaicin **2**.

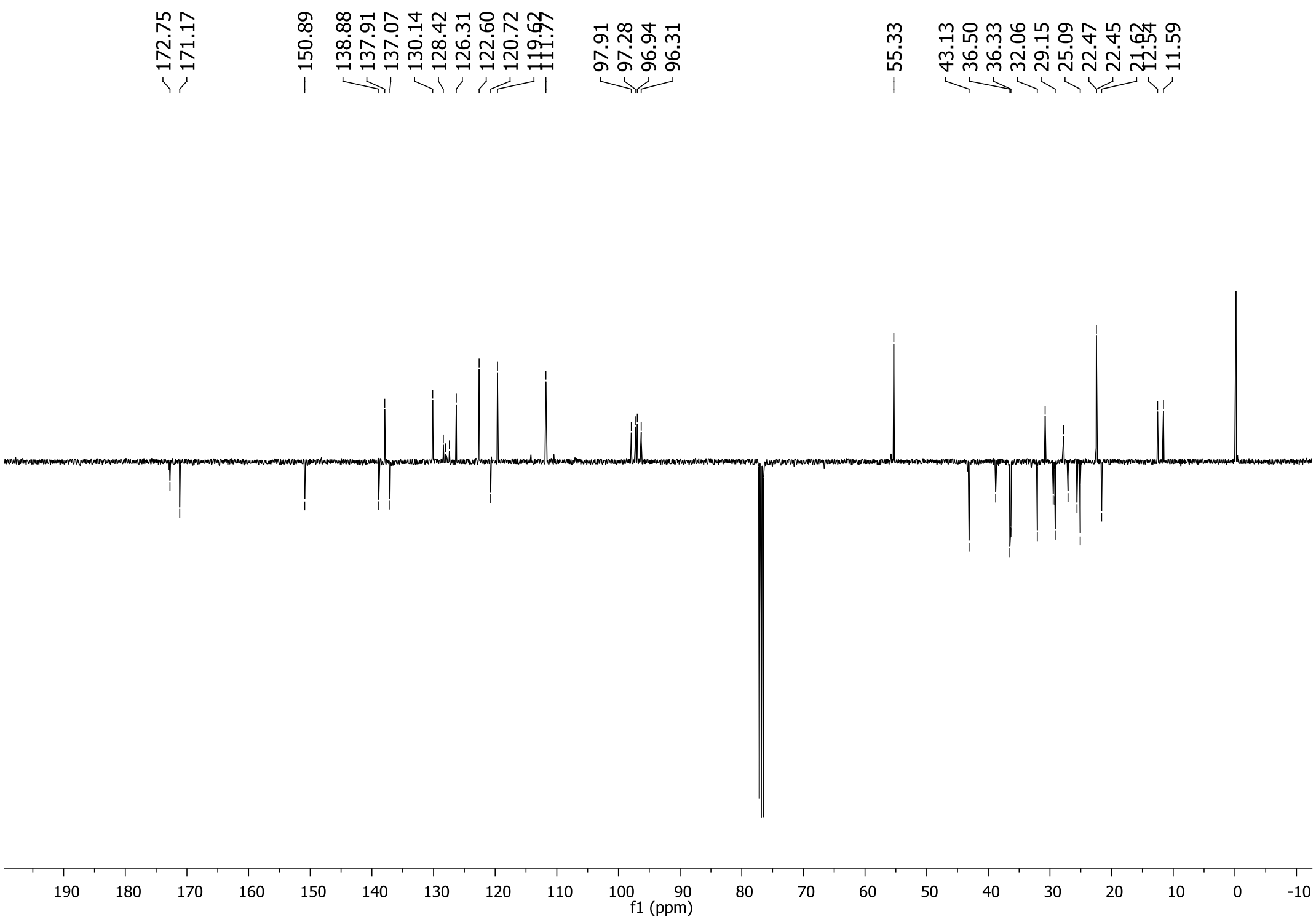

Figure S2. ^{13}C NMR spectrum (APT) (101 MHz, $CDCl_3$) of PpIX-capsaicin **2**.

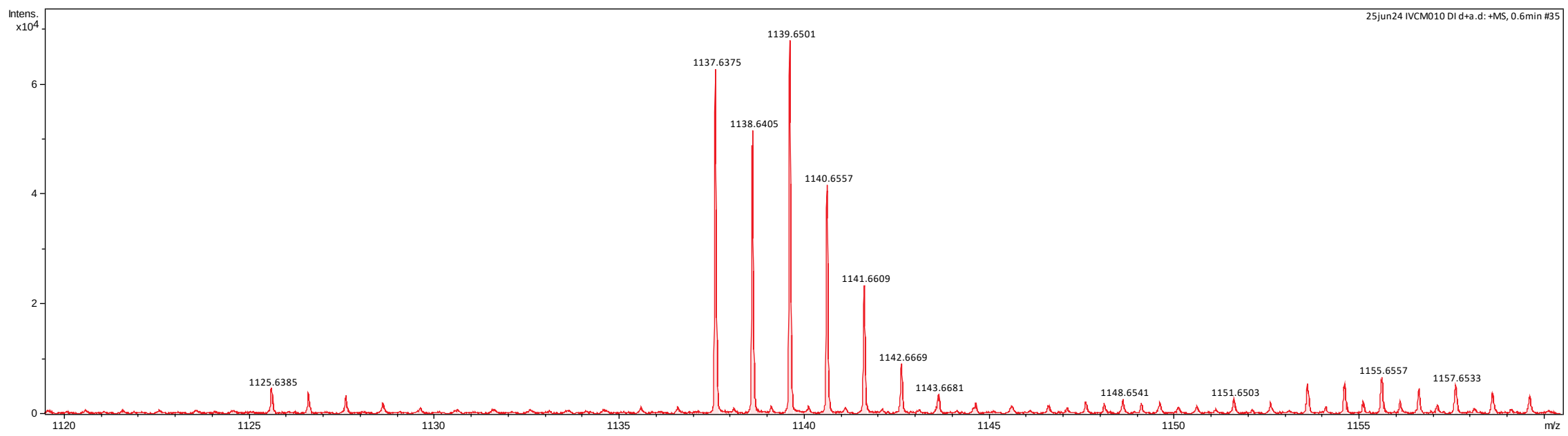

Figure S3. ESI(+)-MS spectrum of PpIX-capsaicin **2** showing the molecular ion peak at m/z corresponding to $[M+H]^+$.

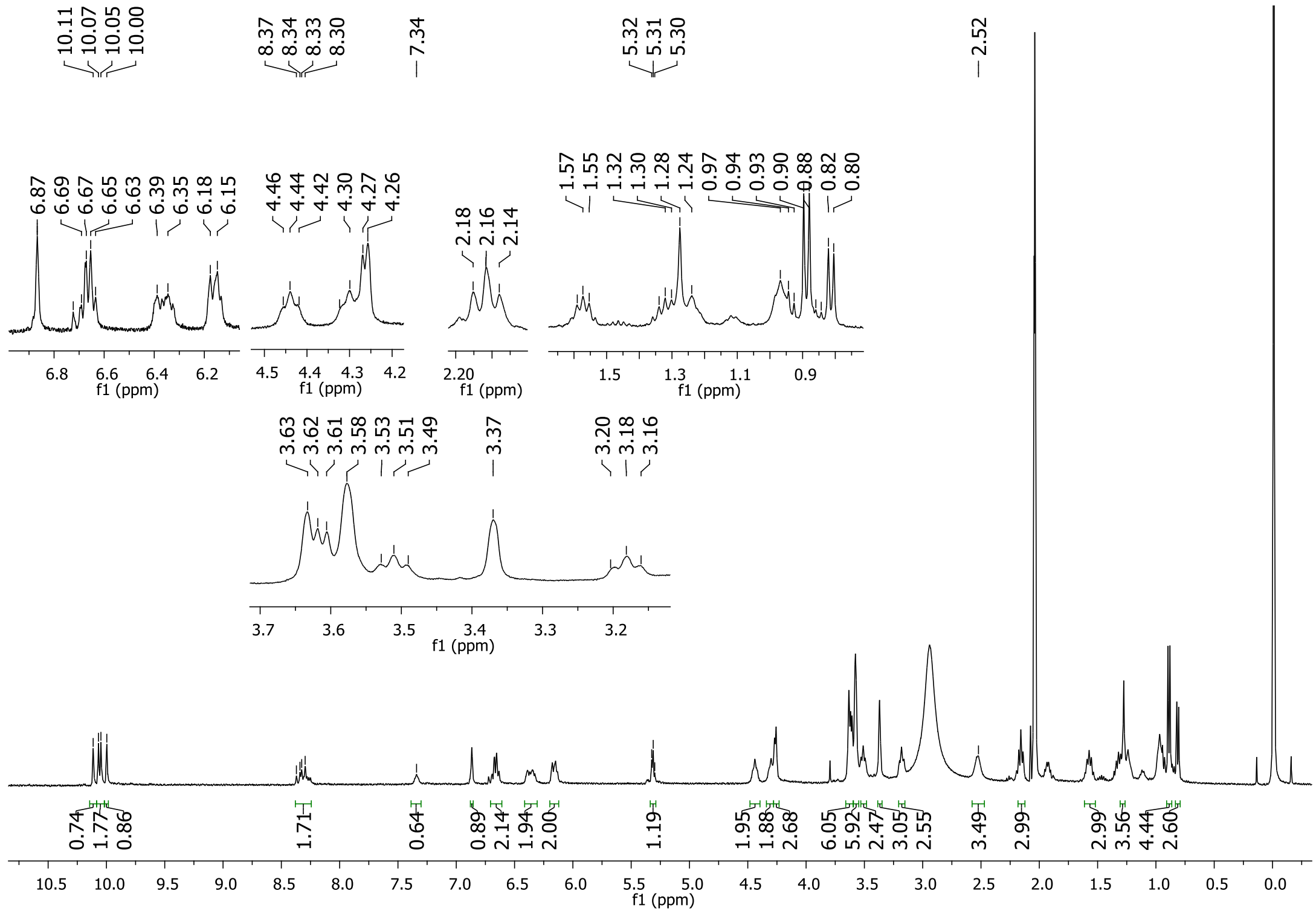

Figure S4. ^{1}H NMR spectrum (400 MHz, $(CD_3)_2CO$) of PpIX-capsaicin **1**.

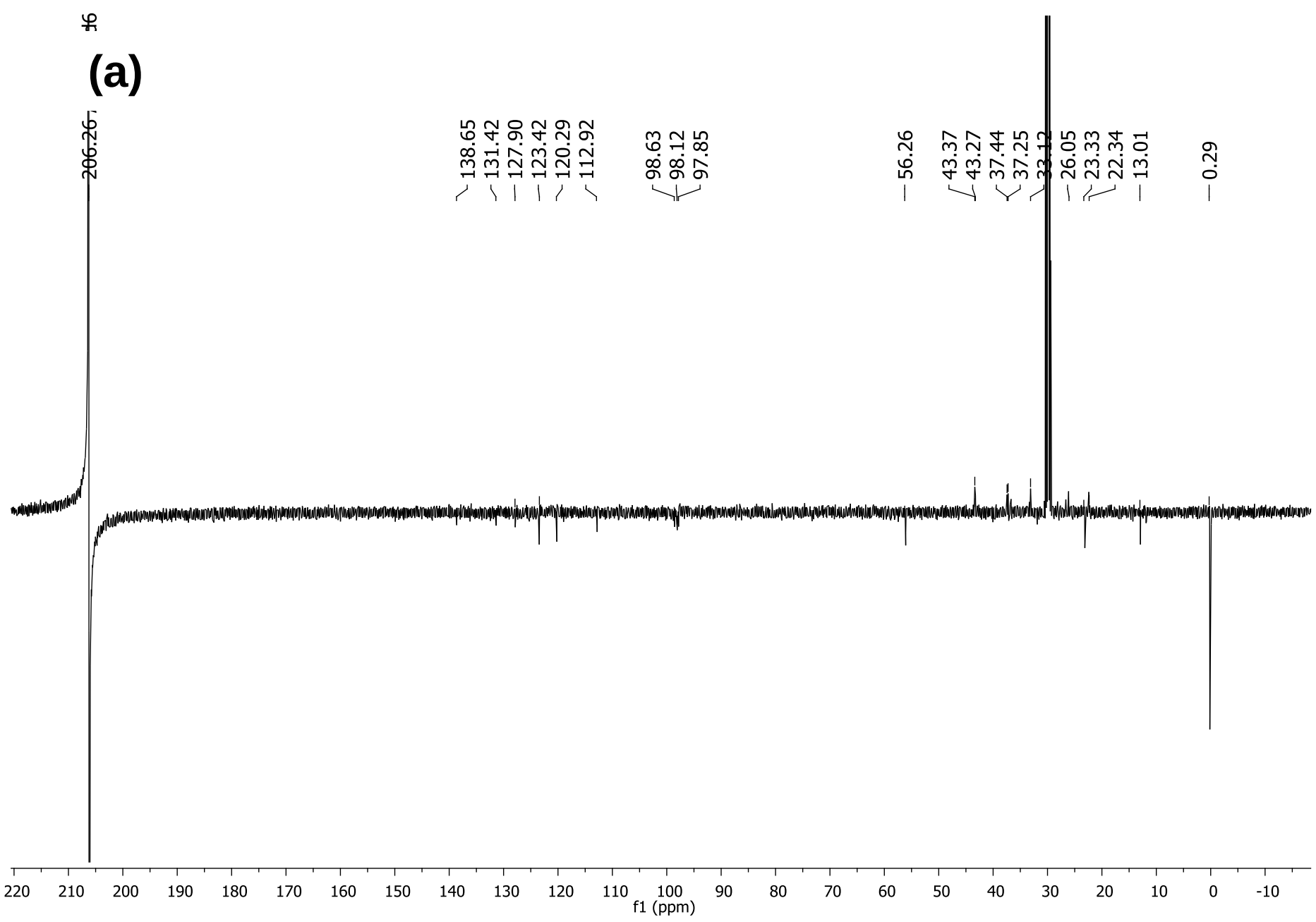

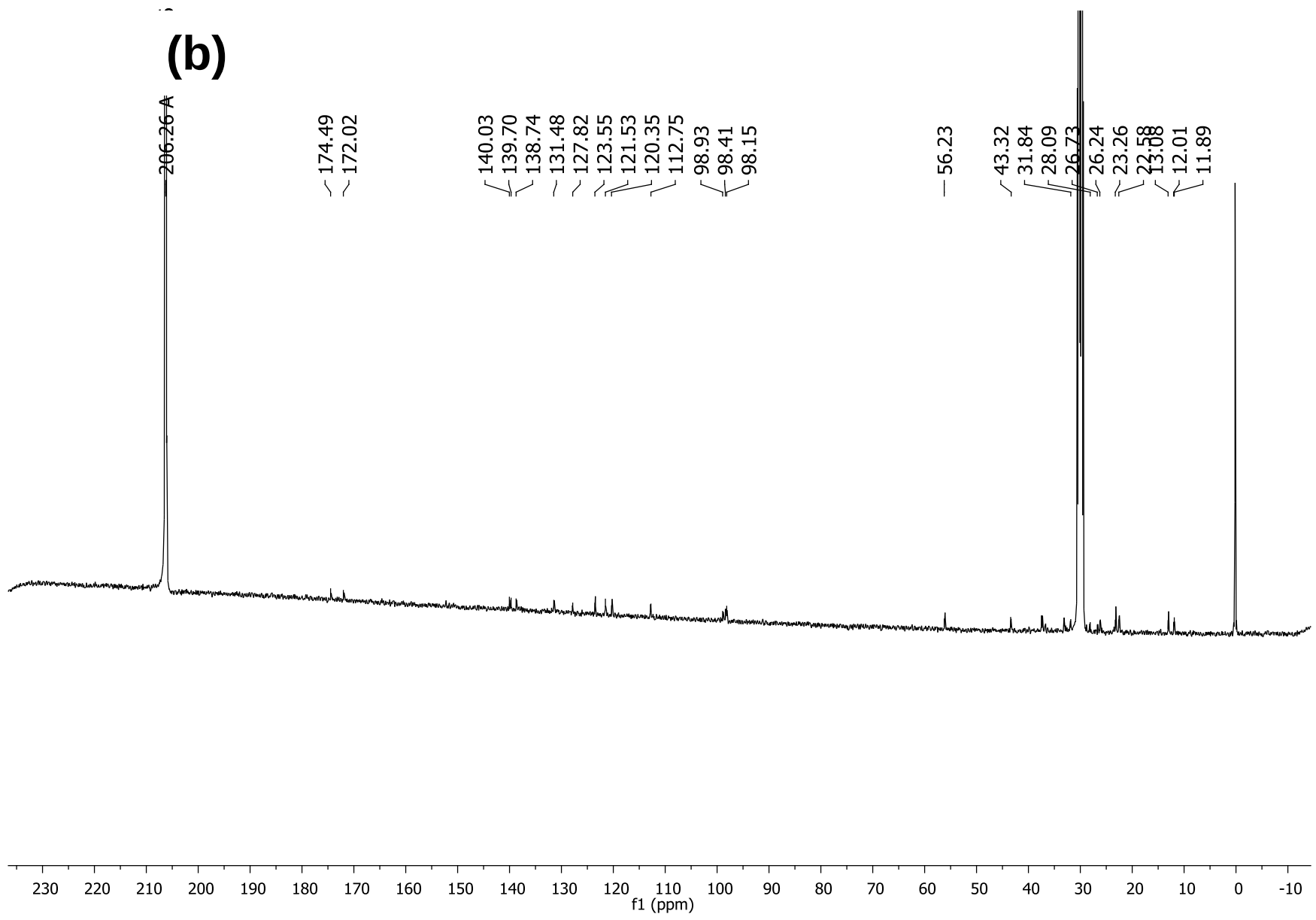

Figure S5. (a) ^{13}C NMR spectrum (APT) and **(b)** ^{13}C NMR spectrum (decoupled) (101 MHz, $CDCl_3$) of PpIX-capsaicin **1**.

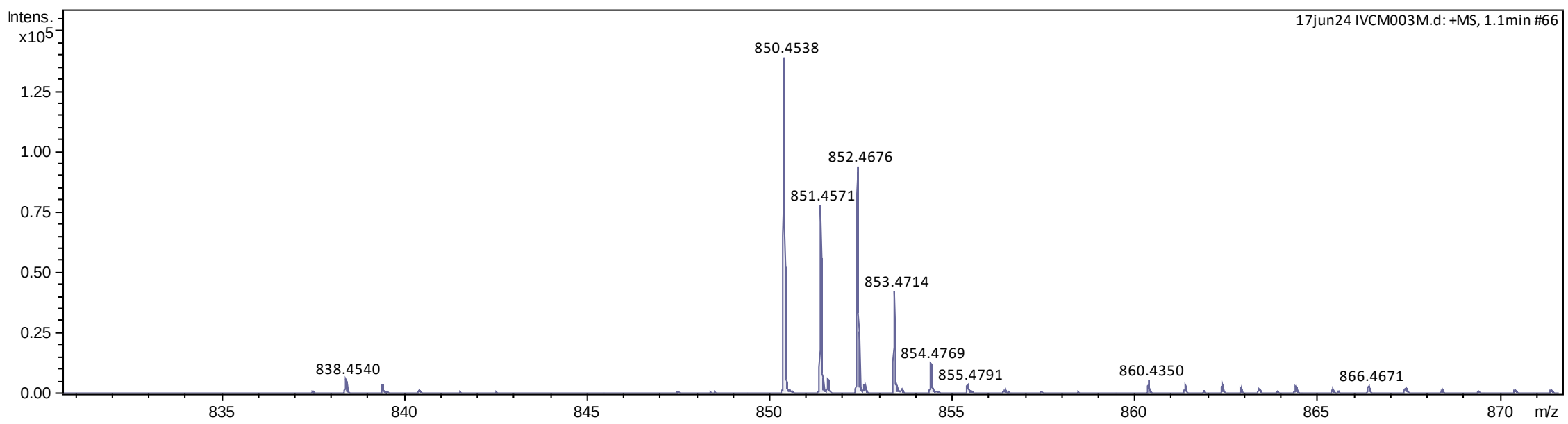

Figure S6. ESI(+)-MS spectrum of PpIX-capsaicin **1** showing the molecular ion peak at m/z corresponding to $[M+H]^+$.

Figure S7. Chemical environments of the hydrogen atoms in capsaicin, and dihydrocapsaicin, attributed to the chemical shifts observed in the ^1H NMR spectrum (values listed in Table S1).

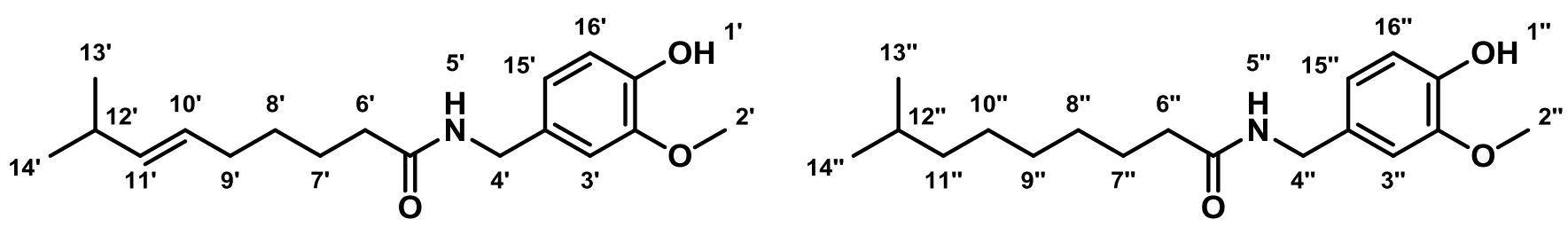

capsaicin

dihydrocapsaicin

Figure S7. Chemical environments of the hydrogen atoms in capsaicin, and dihydrocapsaicin, attributed to the chemical shifts observed in the ^1H NMR spectrum (values listed in Table S1).

Table S2. ^1H chemical shifts (δ_H) of capsaicin (60% purity) reported in the literature (Oliveira Júnior, 2019), compared with those observed for PpIX-capsaicin **2** (400 MHz, $CDCl_3$). Expanded regions of the spectrum are shown in Figure S1, and signals highlighted in bold indicate chemical shifts specific to each capsaicinoid.

Capsaicin	Dihydrocapsaicin

δ H [a] (m[b], J [c])				δ H [a] (m[b], J [c])	
Oliveira Júnior, 2019		PpIX-capsaicin **2** ($CDCl_3$)	*Oliveira Júnior*, 2019		PpIX-capsaicin **2** ($CDCl_3$)
16’	6,85 (1H, d, 8,0)	6,64 (d, 8,0)	15”	6,86 (1H, d, 8,0)	6,64 (d, 8,0)
3’	6,79 (1H, d, 1,8)	6,68 (s)	3”	6,81 (1H, d, 8,0)	6,68 (s)
15’	6,74 (1H, dd, 8,0, 1,8)	6,58 (d, 8,0)	16”	6,76 (1H, dd, 8,0, 1,5)	6,58 (d, 8,0)
11’	**5,90 (2H, m)**	5,92 - 5,91 (m)	4”	4,35 (2H, d, 6,0)	4,23 (d, 5,6)
10’	**5,33 (2H, m)**	5,40 - 5,24 (m)	2”	3,88 (3H, s)	3,69 (s)
4’	4,33 (2H, d, 5,6)	4,23 (d, 5,6)	6”	2,19 (2H, t, 7,5)	2,17 – 2,22 (m)
2’	3,85 (3H, s)	3,69 (s)	7”	1,65 (2H, m)	1,58 – 1,64 (m)
6’	2,20 (2H, m)	2,12 (t, 7,6)	12”	**1,50 (1H, m)**	1,47 – 1,52 (m)
12’	2,20 (1H, m)	2,20 - 2,14 (m)	9” 10” 11”	**1,32-1,26 (6H, m)**	1,22 – 1,30 (m)
9’	**1,98 (2H, dt, 7,5, 6,7)**	1,96 (dd, 13,8, 6,9)	8”	**1,15 (2H, m)**	1,12 – 1,14 (m)
7’	1,64 (2H, tt, 7,5)	1,58 – 1,64 (m)	13” 14”	**0,85 (6H, d, 6,5)**	0,85 (d, 6,6)
8’	**1,37 (2H, tt, 7,5)**	1,30 – 1,38 (m)			
13’ 14’	**0,95 (6H, d, 6,7)**	0,94 (d, 6,7)			

[a] Chemical shifts in ppm, referenced to TMS as the internal standard.
[b] Multiplicity.
[c] Coupling constants in Hz.

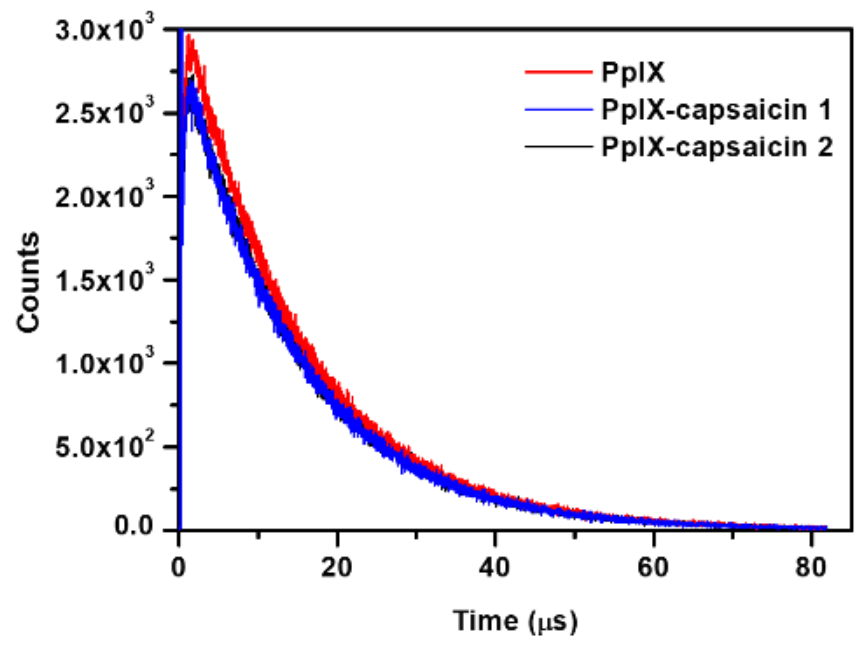

Figure S8. $^1O_2$ decay profile illustrates how the phosphorescence signal intensity at 1270 nm.

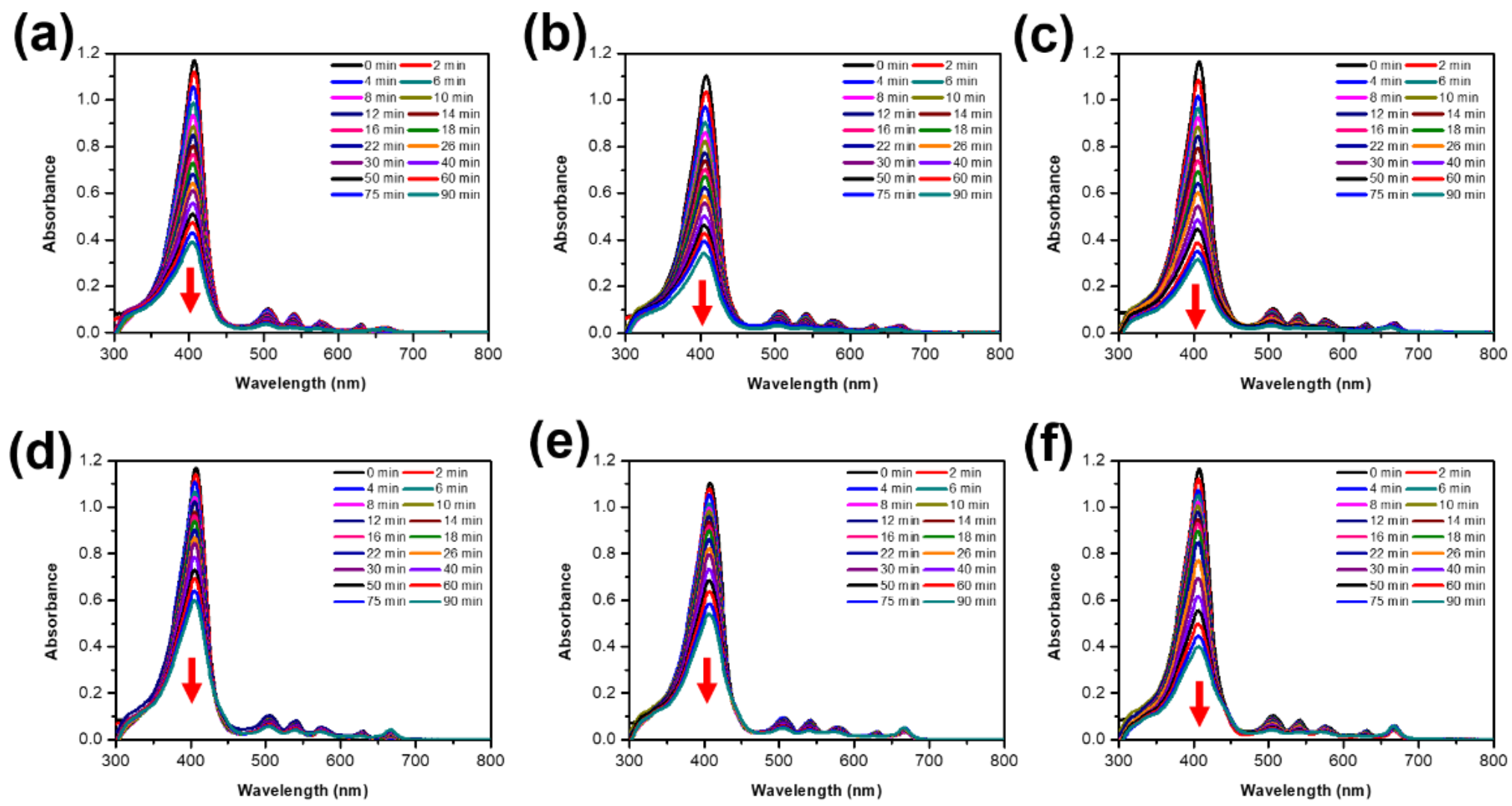

Figure S9. Spectra obtained during photodegradation under white LED irradiation (14.1 mW cm^{-2}, 10 cm from the sample) and green LED irradiation (463.5 μW cm^{-2}, 15 cm from the sample), recorded over 90 min. Panels (a–c) correspond to white LED irradiation of (a) PpIX, (b) PpIX-capsaicin **1**, and (c) PpIX-capsaicin **2**, while panels (d–f) correspond to green LED irradiation of (d) PpIX, (e) PpIX-capsaicin **1**, and (f) PpIX-capsaicin **2**, all in DMSO.

Table S1. UV-Vis absorption spectra recorded during the protonation titration of PpIX and PpIX-capsaicin **1** (c = 16 × 10^{-6} M).

Spectra	Volume of HCl-MeOH (μL)	[HCl] (mol L^{-1})	Volume in the cuvette (μL)	n HCl (mol)	C [H$^+$] (mol L^{-1})	pH
1	4	0.01	2004	0.04	0.00002	4.70
2	2	0.01	2006	0.06	0.00003	4.52
3	2	0.01	2008	0.08	0.00004	4.40
4	2	0.01	2010	0.1	0.00005	4.30

5	4	0.01	2014	0.14	0.00007	4.16
6	2	0.1	2016	0.34	0.00017	3.78
7	5	0.1	2021	0.84	0.00042	3.38
8	5	0.1	2026	1.34	0.00066	3.18
9	2	1.0	2028	3.34	0.00165	2.78
10	2	1.0	2030	5.34	0.00263	2.56
11	2	1.0	2032	7.34	0.00361	2.44
12	5	1.0	2037	12.34	0.00606	2.22
13	5	1.0	2042	17.34	0.00849	2.07
14	10	1.0	2052	27.34	0.01332	1.87
15	2	2.5	2054	32.34	0.01574	1.80
16	15	2.5	2069	69.84	0.03375	1.47
17	15	2.5	2084	107.34	0.05151	1.29
18	50	2.5	2134	232.34	0.10887	0.96
19	50	2.5	2184	357.34	0.16362	0.79
20	100	2.5	2284	607.34	0.26591	0.57
